# GSDMD knockdown attenuates phagocytic activity of microglia and exacerbates seizure susceptibility in TLE mice

**DOI:** 10.1186/s12974-023-02876-w

**Published:** 2023-08-23

**Authors:** Xiaoxia Yang, Qingqing Cao, Yi Guo, Jingchuan He, Demei Xu, Aolei Lin

**Affiliations:** 1 Department of Neurology, Tianjin Neurological Institute, Tianjin Medical University General Hospital, Anshan Road No. 154, Tianjin, 300052 China; 2 Department of Neurology, Bishan Hospital of Chongqing Medical University, Bishan Hospital of Chongqing, No. 9 Shuangxing Road, Chongqing, 402760 China; 3 Department of Neurology, Sichuan Provincial People’s Hospital, University of Electronic Science and Technology of China, 32# W. Sec 2, 1st Ring Rd, Chengdu, 610072 Sichuan China; 4 Department of Otorhinolaryngology Head and Neck Surgery, Tianjin Huanhu Hospital, No.6 Jizhao Road Jinnan District, Tianjin, 300350 China; 5 Department of Neurology, Chongqing Key Laboratory of Neurology, The First Affiliated Hospital of Chongqing Medical University, 1Youyi Road, Chongqing, 400016 China

**Keywords:** GSDMD, Pyroptosis, Phagocytic activity of microglia, P2Y_12_R, Temporal lobe epilepsy

## Abstract

**Background:**

Temporal lobe epilepsy (TLE) is often characterized pathologically by severe neuronal loss in the hippocampus. Phagocytic activity of microglia is essential for clearing apoptotic neuronal debris, allowing for repair and regeneration. Our previous research has shown that gasdermin D (GSDMD)-mediated pyroptosis is involved in the pathogenesis of TLE. However, whether GSDMD-mediated pyroptosis influences the accumulation of apoptotic neurons remains unclear. Therefore, the present study was designed to investigate whether phagocytic activity of microglia is involved in GSDMD-mediated pyroptosis and the pathogenesis of TLE.

**Methods:**

To establish a TLE model, an intra-amygdala injection of kainic acid (KA) was performed. The Racine score and local field potential (LFP) recordings were used to assess seizure severity. Neuronal death in the bilateral hippocampus was assessed by Nissl staining and TUNEL staining. Microglial morphology and phagocytic activity were detected by immunofluorescence and verified by lipopolysaccharide (LPS) and the P2Y_12_R agonist 2MeSADP.

**Results:**

GSDMD knockdown augmented the accumulation of apoptotic neurons and seizure susceptibility in TLE mice. Microglia activated and transition to the M1 type with increased pro-inflammatory cytokines. Furthermore, GSDMD knockdown attenuated the migration and phagocytic activity of microglia. Of note, LPS-activated microglia attenuated seizure susceptibility and the accumulation of apoptotic neurons in TLE after GSDMD knockdown. A P2Y_12_R selective agonist, 2MeSADP, enhanced the migration and phagocytic activity of microglia.

**Conclusions:**

Our results demonstrate that GSDMD knockdown exacerbates seizure susceptibility and the accumulation of apoptotic neurons by attenuating phagocytic activity of microglia. These findings suggest that GSDMD plays a protective role against KA-induced seizure susceptibility.

**Supplementary Information:**

The online version contains supplementary material available at 10.1186/s12974-023-02876-w.

## Introduction

Temporal lobe epilepsy (TLE) is the most common form of partial and drug-resistant epilepsy and is characterized pathologically by severe hippocampal neuronal loss, gliosis, inflammation and seizures [1–3]. Accumulating evidence indicates that pyroptosis plays an important role in the loss of hippocampal neurons in TLE [4]. Pyroptosis is a new form of gasdermin-mediated inflammatory programmed cell death [5]. Among these gasdermin family proteins, GSDMD and GSDME are the most deeply studied in pyroptotic death, especially GSDMD [6]. When the host is stimulated by a variety of exogenous or endogenous factors, GSDMD is cleaved by upstream cysteine proteases, and the N-terminal pore-forming domain is dissociated from the C-terminal repressor domain [7, 8]. Then, the N-terminus oligomerizes and forms pores in the cell membrane, leading to cell swelling, cell membrane disruption, the release of inflammatory molecules and cell pyroptotic death [9]. Our previous research has shown that GSDMD knockdown exacerbates hippocampal damage in kainic acid (KA)-induced TLE, leading to a transition from pyroptosis to apoptosis and the accumulation of apoptotic cells; as a result, seizure severity is exacerbated [10]. However, the precise mechanism behind the accumulation of apoptotic cells after GSDMD knockdown and their contribution to TLE remain poorly understood.

A prominent event in the development of TLE is the loss of neuronal cells. This event involves programmed cell death, the triggering of which is regulated, and is followed by efficient removal of cell corpses. If neuronal debris is not removed in a timely manner, accumulated debris may influence central nervous system (CNS) function. As professional phagocytes, microglia can rapidly engulf and degrade dead or apoptotic cells and inhibit further inflammation [11]. When challenged with disease or inflammation, microglia resort to different strategies to boost their phagocytic efficiency and compensate for the increased number of apoptotic cells, thus maintaining tightly coupled phagocytic activity and apoptosis [3]. In subsequent studies, it was discovered that this coupling is a pivotal pathway for the clearance of apoptotic cells. Importantly, phagocytic activity of microglia in epilepsy is mainly mediated by the P2Y_12_ receptor (P2Y_12_R), a metabotropic purinergic receptor that is known to control microglial activation and migration by elevating Ca^2+^ from intracellular Ca^2+^ stores; this occurs in response to extracellular environmental changes during physiological brain activity and after pathological events [12]. However, phagocytic activity of microglia and the role of P2Y_12_R in the accumulation of apoptotic cells resulting from GSDMD knockdown are not known.

Thus, the aim of the present study was to explore whether GSDMD-mediated pyroptosis is associated with phagocytic activity of microglia and is involved in the mechanism of TLE. Our results demonstrate that the knockdown of GSDMD attenuates phagocytic activity of microglia by P2Y_12_R and exacerbates hippocampal neuronal injury after administration of KA, thus revealing an uncircumscribed protective role of GSDMD in TLE pathogenesis.

## Materials and methods

### Animals

Male C57BL/6L mice (26–30 g, 8–12 weeks old) were obtained from Charles River Laboratories. This study was conducted in accordance with the National Institutes of Health guidelines for the use of experimental animals. All experiments were approved by the institutional Animal Care and Use Committees of Tianjin Medical University General Hospital. All animals were housed in a temperature-controlled environment in a 12/12 h light–dark cycle and provided free access to food and water.

### Viral vectors and infection

Adeno-associated virus (AAV) serotype 9 expressing hSyn-driven mouse *Gsdmd* shRNA was designed and synthesized by GeneChem (Shanghai, China). An shRNA with a targeting sequence of 5′-TTGATGAGGAGGAATTAAT-3′ directed against *Gsdmd* was used to reduce hippocampal GSDMD levels, and the control shRNA sequence was 5′-CGCTGAGTACTTCGAAATGTC-3′, as described in our previous study [10].

Mice were anesthetized with 2% isoflurane and placed on a stereotaxic apparatus (David Kopf Instruments, Tujunga, CA, USA). Using a 5-μl syringe (Hamilton, Reno, NV), three weeks before the establishment of the TLE model, 0.5 μl of *Gsdmd* shRNA AAV or control shRNA AAV was injected into the bilateral hippocampus separately (AP: − 2.0 mm; ML: ± 1.5 mm; DV: − 1.8 mm from bregma) at a rate of 0.05 μl/min. After each infusion was completed, the needle was held in place for 10 min to minimize reflux along the injection trace.

### KA-induced TLE model

The KA model was established 3 weeks after AAV injection. Mice were anesthetized with 2% isoflurane and immobilized in a stereotaxic apparatus. 0.3 µg KA in 0.2 μl artificial cerebrospinal fluid (aCSF) was injected into the right basolateral amygdala nucleus (AP: − 0.94 mm; ML: + 2.85 mm; DV: − 3.75 mm from bregma) as previously described [10, 13]. One hour after KA injection, lorazepam (6 mg/kg, Merck, MO, USA) was administered intraperitoneally to stop the seizures and reduce morbidity and mortality. The Racine scale was used to evaluate seizure severity [10]. Mice that presented with seizures greater than or equal to Racine stage 4 were evaluated to determine the number of SRSs and the latency period.

### Intrahippocampal administration of LPS

LPS treatment was given 24 h before KA administration. 1.0 mg/kg LPS (*E. coli* stereotype 055:B5, Sigma-Aldrich, St-Louis, MO, USA) was diluted to 1 μg in 1 μl of 0.9% of sterile saline as previously described [14]. Briefly, mice were anesthetized, weighed, and placed on a stereotaxic apparatus. 5 µl LPS was injected into the bilateral hippocampus (AP: − 2.0 mm; ML:  ± 1.5 mm; DV: − 1.8 mm from bregma) at a rate of 1 μl/min over 5 min by an electronic Stereotaxic Injector. The accuracy of the LPS injection to the bilateral hippocampus was confirmed by periodic injection of Evan’s blue dye. Animal body weights were measured, after which mice were euthanized at 28 days following surgeries.

### Electrode implantation and local field potential recording

As we previously described, implantation and local field potential (LFP) recordings were performed one week before KA injection [10]. Two stainless steel screws were implanted in the frontal cortex as ground screws, and platinum iridium alloy microwire was implanted into the right hippocampus (AP: − 2.0 mm; ML: + 1.5 mm; DV: − 1.8 mm from bregma). The microwire was cemented to the skull as well as a U-shaped frame to hold the head.

Twenty-eight days after KA injection, LFP recordings were performed for 2 h as previously described. The head of the conscious mouse was fixed via a U-shaped frame to minimize behavioral state-induced LPF signal changes. LFP signals were recorded using an MAP data acquisition system (Plexon, Dallas, TX, USA), filtered at 0.1 ~ 500 Hz, preamplified and digitized at 4 kHz. The LFP signals were analyzed using NeuroExplorer (Nex Technologies, Littleton, MA, USA). A cluster of spontaneous paroxysmal discharges with high frequencies (> 5 Hz), high amplitudes (> 2 times the baseline), and long durations (> 5 s) were defined as seizure-like events (SLEs) [15].

### Nissl staining

Serial transverse sections made from brain tissue of mice embedded in a paraffin block were dewaxed, rehydrated, and immersed in 0.1% toluidine blue (Servicebio, Wuhan, China) at 50 °C for 5 min. After washing in water, the sections were dehydrated in 95% ethanol, cleared in xylene, and coverslipped with neutral balsam. The sections were examined under a light microscope. The number of surviving neurons was confirmed by the exhibition of Nissl substance, euchromatic nucleus, and nucleolus. Then, the density of surviving neurons was calculated using ImageJ as previously described (26). For each animal, approximately six sections containing the hippocampus (− 1.3 ~ − 3.5 AP) were randomly selected.

### Immunofluorescence staining

As we previously described [10, 16], brains removed from test mice were frozen and later sectioned into slices with 20 μm thickness before fixation with 4% paraformaldehyde for 30 min. The sections were incubated with 5% goat serum and 0.4% Triton X-100 for 20 min and then incubated with primary antibodies against ionized calcium binding adapter molecule 1 (Iba-1) (1:200; NB100-1028, Novus Biologicals, CO, USA), glial fibrillary acidic protein (GFAP) (1:200; ab4674, Abcam, Cambridge, MA, USA), CD206 ( 1:400, 24595, Santa Cruz, CA, USA) and B7-2(1:100, 91882, Santa Cruz, CA, USA) overnight at 4 °C. After being washed with PBS three times for 5 min each, the sections were incubated with secondary antibodies (1:1000) for 1 h at room temperature. Images were captured by confocal microscopy (Leica, Wetzlar, Germany) or fluorescence microscopy (Olympus, model BX-61, Tokyo, Japan).

To assess cell death, brain sections were stained with terminal deoxynucleotidyl transferase-mediated dUTP nick end labeling (TUNEL) using an in situ cell death detection kit (Roche, Basel, Switzerland), as we performed elsewhere [17]. TUNEL staining was performed according to the protocol provided by the manufacturer. Cell nuclei were labeled with 4′,6-diamidino-2-phenylindole, dihydrochloride (DAPI). Images were captured by microscopy. Image analysis was performed using ImageJ software (National Institutes of Health, MD, USA).

### Western blotting

Brain tissues and cells were lysed in RIPA lysis buffer (Sigma-Aldrich, St. Louis, MO, USA) supplemented with PMSF (Sigma-Aldrich, St. Louis, MO, USA). As previously described [18, 19], equal amounts of denatured protein were separated by SDS-PAGE and transferred to polyvinylidene difluoride membranes (Millipore, Billerica, MA, USA). The membranes were blocked in 5% nonfat dry milk in Tris-buffered saline with Tween (TBST) and then incubated with one of the following primary antibodies: anti-P2Y_12_R (ab184411, Abcam, Cambridge, MA, USA). The blots were then incubated with horseradish peroxidase-conjugated anti-rabbit secondary antibodies (1:5000, ZB-2305, ZB2301, Zhongshan Golden Bridge, Beijing, China) and developed using the enhanced chemiluminescence system. GAPDH was used as an internal control.

### Real-time RT-PCR

Total RNA was extracted from the substantia nigra and striatum with TRIzol reagent (Thermo-Fisher Scientific, Waltham, MA, USA), as we previously described[20, 21]. The cDNA was transcribed with a Trans-Script First-Strand c-DNA Synthesis SuperMix Kit (TransGen Biotech, Beijing, China) according to the manufacturer’s instructions. PCR was performed on an Opticon 2 Real-Time PCR Detection System (Bio-Rad, Hercules, CA, USA) with corresponding primers (Table 1) and SYBR green PCR Master Mix (Roche Diagnostics, Basel, Switzerland). The CT values for triplicate samples were averaged, and the data were analyzed with the ΔΔCT method, where fold change = 2^−ΔΔCT^. Expression levels of the mRNAs were then reported as fold changes vs. control. Quantitative levels of mRNAs were normalized to β-actin expression.

**Table 1:** Primer sequences for quantitative RT-PCR

Gene	Primer, 5′–3′	
	Forward	Reverse
CD74	AGTGCGACGAGAACGGTAAC	CGTTGGGGAACACACACCA
IL-6	ACCGCTATGAAGTTCCTCTCTGCA	AAGCCTCCGACTTGTGAAGTGGT
IL-1β	GCTGCTTCCAAACCTTTGAC	AGCTTCTCCACAGCCACAAT
TNF-α	ACGGCATGGATCTCAAAGAC	GTGGGTGAGGAGCACGTAGT
CD163	ATGGGTGGACACAGAATGGTT	CAGGAGCGTTAGTGACAGCAG
IL-4	GCAACGAAGAACACCACAGA	TGCAGCTCCATGAGAACACT
IL-10	AAATAAGAGCAAGGCAGTGG	AAATAAGAGCAAGGCAGTGG
TGF-β	CTGTACATTGACTTCCGCAAG	TGTCCAGGCTCCAAATGTAG

### Cell culture

*Primary hippocampal neuronal culture* Hippocampal tissue was dissected from embryonic day 18 (E18) C57L embryos under a 10 × microscope. The hippocampal tissue was then minced with scissors in ice-cold neurobasal medium (Invitrogen, USA). Thereafter, minced tissue was digested with papain (20 U/mg; Worthington) at 30 °C for 20 min in tubes shaken at 120 rpm in a water bath. After digestion, the reaction was stopped by adding inactivated fetal bovine serum. After trituration and centrifugation, cells were resuspended in Dulbecco’s modified Eagle’s medium (DMEM) supplemented with 10% fetal bovine serum and then seeded in polylysine (Sigma, USA)-coated 12-well plates at 5 × 10^5^ cells per well. Then, the medium was replaced by serum-free Neurobasal^®^ medium containing 2% B27 medium-supplement (GIBCO, USA), 0.5 mM glutamine and antibiotics 4 h later. The cultures were essentially free of astrocytes and microglia and maintained at 37 °C in a humidified incubator with 5% CO_2_. Half of the culture media was changed every 3 days. The neurons were used for experiments after 7 days.

*N9 cells culture* N9 cells were obtained from the American Type Culture Collection (ATCC, Manassas, VA, USA). N9 cells were cultured in DMEM supplemented with 10% fetal bovine serum, 100 U/ml penicillin, 100 μg/ml streptomycin and 200 mM GlutaMAX™ Supplement (GIBCO, Grand Island, NY, USA). Cells were plated at a density of 2 × 10^6^ cells/insert.

*Transwell coculture of hippocampal neurons and N9 cells* Primary hippocampal neurons were treated with either *Gsdmd* shRNA or the control shRNA at 2 days in vitro (DIV2) for 24 h and then exposed to KA (100 µM) or DMEM for an additional 12 h at DIV7 [22]. N9 cells were activated with lipopolysaccharide (LPS, 100 ng/mL; Sigma) for 6 h, which is commonly used to activate microglia without cytotoxicity. In addition, N9 cells were activated with P2Y_12_R agonist 2MeSADP (100 µM; Sigma) for 24 h, which can activate phagocytic activity of microglia. After 48 h of separate growth, N9 cells and purified hippocampal neurons (Chinese Academy of Sciences, Kunming, China) were seeded onto 24-well plates and Transwell permeable support membrane inserts (0.4 μm, Corning, USA), respectively. After the coculture was finished, the exposed hippocampal neurons or N9 cells were washed with PBS and harvested for further phagocytic activity assays or protein analysis and immunofluorescence staining.

*Crystal violet staining* After 48 h of cultivation of the Transwell system, the upper chamber was washed with PBS three times, and the unmigrated cells were wiped off with cotton. Then, we fixed the cells using 4% paraformaldehyde for 20 min and subsequently stained them with 0.1% crystal violet for 5 min. Finally, the cells were washed and counted under a microscope. All experiments were repeated independently three times.

*Phagocytosis assay* pH-sensitive green fluorophore-tagged Escherichia coli (*E. coli*) bioparticles (pHrodo Green *E. coli* BioParticles Conjugates, #P35366, Invitrogen Corporation, Frederick, MD, USA) were used to measure phagocytic activity. N9 microglia (5 × 10^5^/well) were plated in 6-well plates and cultured for 24–72 h. A total of 100 μg of pHrodo Green *E. coli* BioParticles Conjugates were added per condition and incubated with N9 cells for 30 min at 37 °C. Phagocytosis was inhibited with 10 μM cytochalasin D (Cyto. D; #SI-C8273, Sigma‒Aldrich), which was added 30 min before the addition of PHrodo *E. coli* bioparticles as a negative control. N9 cells were examined under a fluorescence microscope to determine whether the bioparticles had been phagocytosed and were present inside the cells. The number of N9 cells containing more than one bioparticle was counted, and the percentage of pHrodo^+^ cells was determined.

### Whole-cell patch-clamp recording

Whole-cell recordings were prepared as previously described [23]. N9 cells on coverslips were mounted in a recording chamber, and the tissue culture medium was replaced with an extracellular (bath) solution (125 mM NaMeSO_4_, 5 mM KMeSO_4_, 1 mM MgCl_2_, 1 mM CaCl_2_, 5 mM glucose, and 10 mM HEPES, pH 7.4). Whole-cell currents were recorded at room temperature (22 ± 1 °C) and compensated online for series resistance and capacitance. Pipettes (3–4 MΩ resistance) were filled with an intracellular solution (133 mM KMeSO_4_, 2 mM KCl, 2 mM K_2_ATP, 0.9 mM CaCl_2_, 1 mM EGTA, and 10 mM HEPES, pH 7.2) buffered to 1.0 μm free Ca^2+^. These low-Cl^−^ solutions eliminated the swelling-activated Cl − current. For KCa3.1 recordings, N9 cells were held at − 80 mV. Then, the cells were stimulated with a step protocol of 400 ms duration, ranging from − 120 to + 60 mV every 5 s for up to 5 min. Recordings were made with a Multiclamp 700B amplifier, and data were digitized with a Digidata 1440A and pClamp 10.3 software (Axon Instruments, CA, USA). The results were analyzed with Clampfit 10.3 software.

### Statistical analysis

Data are expressed as the mean ± SD. Two-tailed unpaired Student’s *t* test was used to determine the significance of differences between two groups. One-way ANOVA followed by Tukey’s post hoc test was used for more than 2 groups. Two-way repeated ANOVA followed by Bonferroni posttests was performed for multiple comparisons. *P* < 0.05 was considered statistically significant. GraphPad Prism software was used for the statistical analyses.

## Results

### Knockdown of GSDMD augments the accumulation of apoptotic neurons and seizure susceptibility in TLE mice

**Fig. 1:**
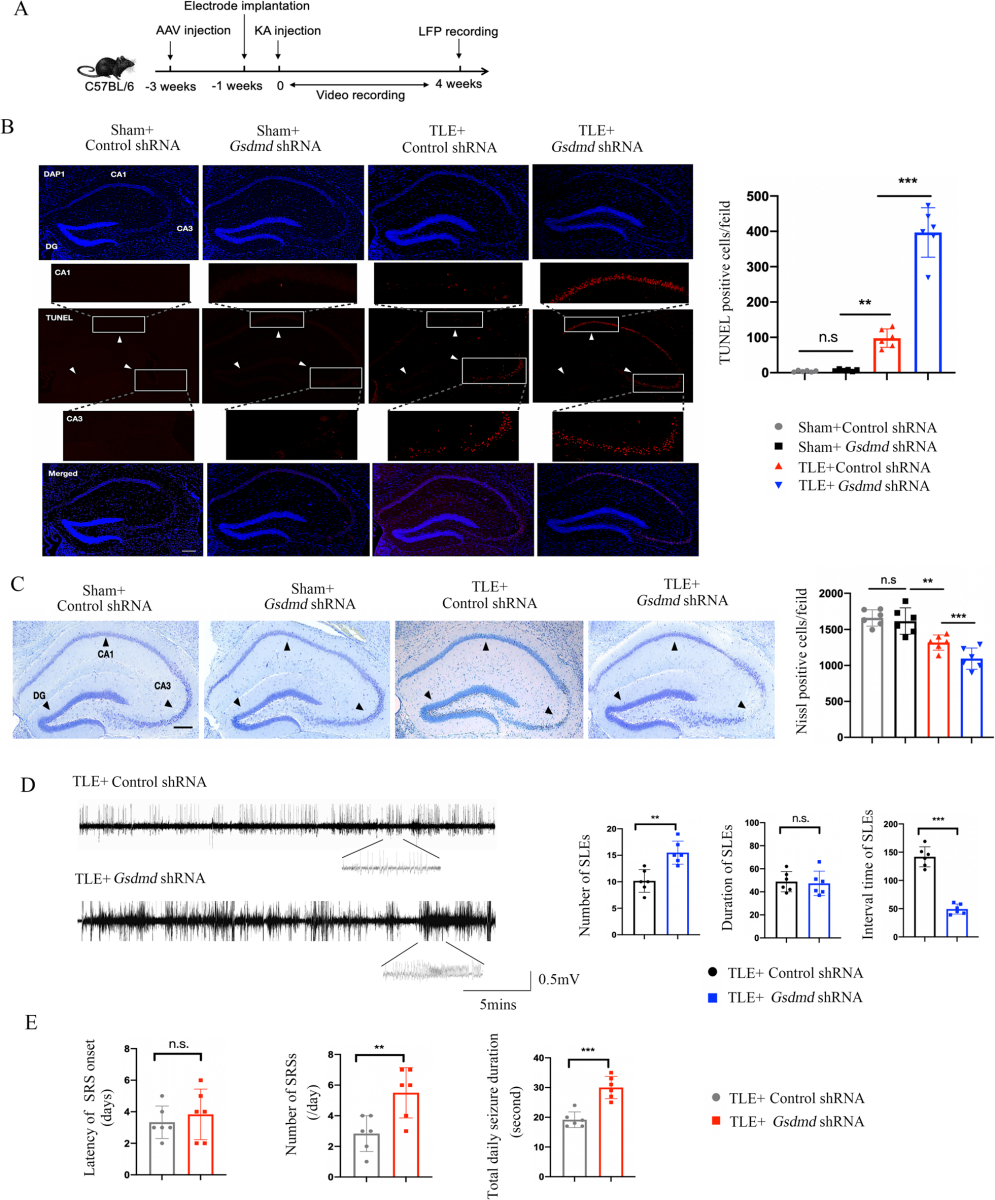
GSDMD knockdown increases the accumulation of apoptotic neurons and seizure susceptibility in TLE mice. **A** Schematic showing the experimental design. **B** GSDMD knockdown’s effect on TUNEL staining in brain sections containing the hippocampus from mice with KA-induced TLE at 28 days. Scale bar = 100 µm. The bar graph shows the effect of GSDMD knockdown on the number of TUNEL^+^ cells in mice with KA-induced TLE. **C** Representative images of Nissl staining of subregions of the hippocampus at 28 days after GSDMD knockdown in mice with KA-induced TLE. Scale bar = 100 µm. The bar graph shows quantitative analysis of the number of Nissl^+^ cells in the hippocampus. **D** Representative LFP signals in the TLE + control shRNA and TLE + *Gsdmd* shRNA groups. The bar graphs show quantitative analysis of the number, duration, and interval of SLEs. **E** Quantitative analysis of the latency and number of SRSs and total daily seizure duration. *n* = 6 mice per group. All data are presented as the mean ± SD; *P < 0.05, **P < 0.01, and ***P < 0.001

Our previous research has shown that GSDMD-mediated pyroptosis is involved in the pathogenesis of TLE and that GSDMD is expressed mainly in hippocampal neurons [10]. The efficiency of GSDMD knockdown was confirmed by western blot 28 days after KA injection, and no change in GSDMD was found after control shRNA injection (Additional file 1: Fig. S1A). To determine the effect of GSDMD knockdown on KA-induced hippocampal neuronal loss, we evaluated the extent of cell death by measuring the numbers of TUNEL-positive cells and evaluated the surviving neurons by Nissl staining at day 28 after vehicle or KA treatment. We found that the numbers of TUNEL-positive cells observed in the hippocampus of mice exposed to KA underwent a statistically significant increase after GSDMD knockdown (Fig. 1B). Conversely, the counts of neurons in the hippocampus (Nissl-positive cells) were significantly reduced in KA-induced TLE mice subjected to GSDMD knockdown (Fig. 1C).

Next, we validated whether GSDMD knockdown could exacerbate seizure activities. After receiving an injection of KA, GSDMD knockdown mice were subjected to 4 weeks (24 h/day) of video recording (Fig. 1A), and all mice developed SRSs after the latency period (3–7 days) as previously described[10]. Seizure activity was recorded using LFPs 4 weeks after KA administration, and we analyzed the SLEs for a period of 30 min from the LFPs. Compared with the control shRNA-treated mice, mice treated with *Gsdmd* shRNA exhibited a significantly increased number of SLEs and shorter intervals (Fig. 1D). Meanwhile, mice treated with *Gsdmd* shRNA exhibited an increased seizure frequency compared with that of control shRNA-treated mice (Fig. 1E). However, there was no significant difference in the latency of SRSs between the two groups (Fig. 1E). Together, these results suggest that GSDMD knockdown augments KA-induced seizure susceptibility and the accumulation of apoptotic neurons.

### GSDMD knockdown reduces the number of microglia and enhances microglial ramification

**Fig. 2:**
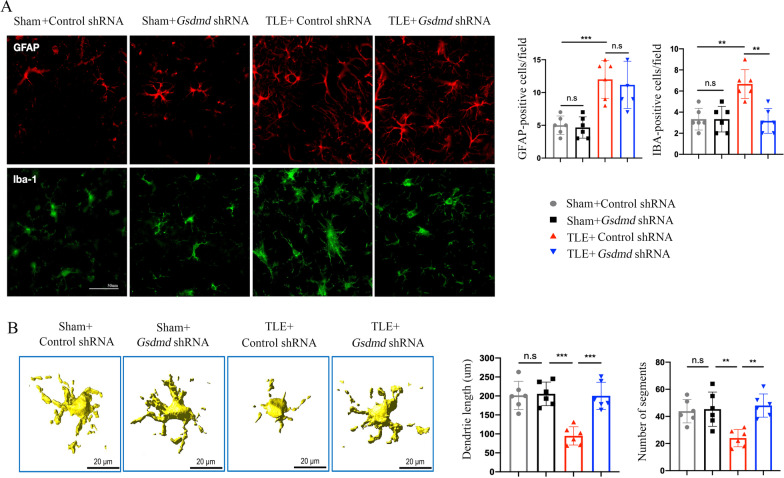
GSDMD knockdown reduces the number of microglia and increases microglial ramification. **A** Immunofluorescence staining of astrocytes (GFAP, red) and microglia (Iba1, green) in the CA1 region of hippocampus of sham and TLE model mice that received *Gsdmd* shRNA or control shRNA at day 28. The bar graphs show the number of astrocytes and microglia in the hippocampus of sham and TLE mice at day 28. **B** Representative Imaris-based three-dimensional reconstructed images of Iba-1^+^ microglia from sham and TLE model mice that received *Gsdmd* shRNA or control shRNA. The bar graphs show the morphological features of microglia, including dendrite length and the number of segments, which were quantified using Imaris. *n* = 6 mice per group. All data are presented as the mean ± SD; *P < 0.05, **P < 0.01, and ***P < 0.001

Apoptotic neurons must be quickly removed to avoid the further toxic effects they exert in the hippocampus, a process executed by phagocytes, including microglia and astrocytes. To determine the underlying mechanism by which GSDMD knockdown triggered apoptosis, we measured the counts of microglia and astrocytes. We found that the number of microglia decreased significantly after GSDMD knockdown compared with the control shRNA group in KA-treated mice, while the number of astrocytes did not change significantly (Fig. 2A), which suggested that the phagocytic activity of microglia played an important role after GSDMD knockdown.

Then, we assessed the morphology of microglia from the hippocampus of TLE or sham mice using confocal microscopy, and the images were reconstructed in three dimensions by Imaris software. The processes of microglia in the GSDMD knockdown mice were more ramified than those from the control shRNA mice (Fig. 2B). Moreover, the altered morphology was verified by increased bud-like extensions and number of segments in the GSDMD knockdown mice (Fig. 2B). Therefore, GSDMD knockdown has been demonstrated to effectively maintain the process by which microglia branch; as a result, microglia remain in their resting state, which may enhance their monitoring function but reduce their phagocytic function.

### GSDMD knockdown promotes microglial M1 polarization and local inflammation

**Fig. 3:**
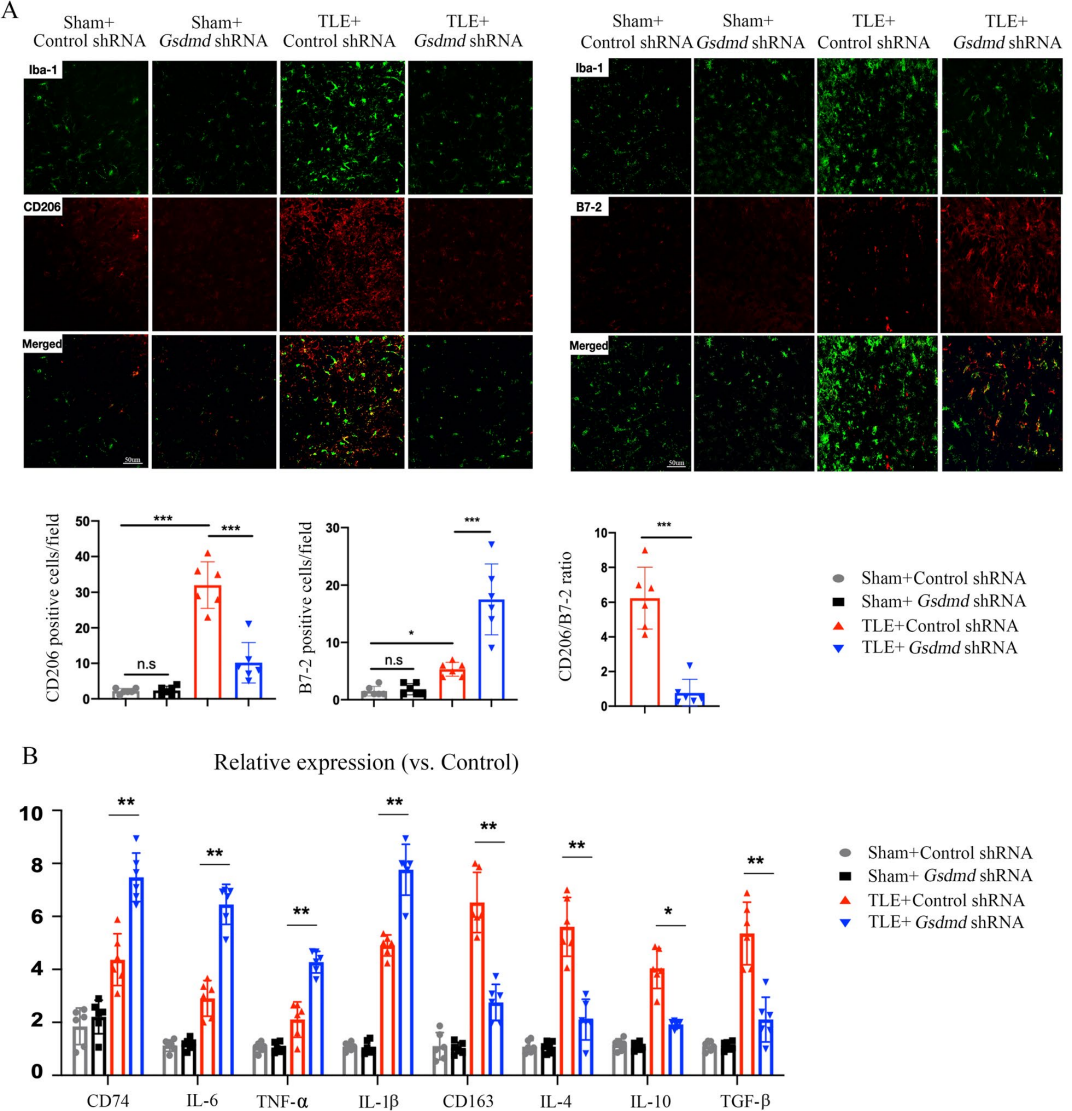
GSDMD knockdown promotes microglial M1 polarization and local inflammation. **A** Immunostaining images of M1 microglia (Iba1^+^ B7-2^+^) and M2 microglia (Iba1^+^ CD206^+^) in the CA1 region of hippocampus of sham and TLE model mice that received *Gsdmd* shRNA or control shRNA at day 28. The bar graphs show the number of M1 microglia and M2 microglia and the CD206/B7-2 ratio in the CA1 region of hippocampus of sham and TLE model mice at day 28. **B** The bar graphs show the mRNA expression of CD74, IL-6, TNF-α, IL-1β, CD163, IL-4, IL-10, and TGF-β in hippocampal tissues from mice that received the indicated treatment. *n* = 6 mice per group. All data are presented as the mean ± SD; *P < 0.05, **P < 0.01, and ***P < 0.001

Next, we explored microglial polarization and inflammatory cytokine production in TLE mice after GSDMD knockdown. Brain sections were immunostained for B7-2 (M1-like marker) or CD206 (M2-like marker). Activated microglia were defined based on cell morphology and a cell body diameter cutoff of 7.5 μm [24]. At KA treatment was performed for 4 weeks, we observed that the number of CD206 microglia was increased in control shRNA-treated mice and that the number of B7-2 microglia was increased in *Gsdmd* shRNA-treated mice (Fig. 3A). Moreover, we collected brain tissue from the hippocampus to determine the levels of M1- and M2-associated inflammatory cytokines. GSDMD knockdown significantly downregulated the expression of CD163 (M2-like marker) and anti-inflammatory cytokines, such as IL-4, IL-10, and TGF-β, and upregulated the expression of CD74 (M1-like marker) and pro-inflammatory cytokines, including IL-1β, IL-6 and TNF-α in TLE mice (Fig. 3B); these results suggest that GSDMD knockdown promotes microglial M1 polarization from M2 and augments KA-induced local inflammation.

### GSDMD knockdown attenuates the migration and phagocytic activity of microglia

**Fig. 4:**
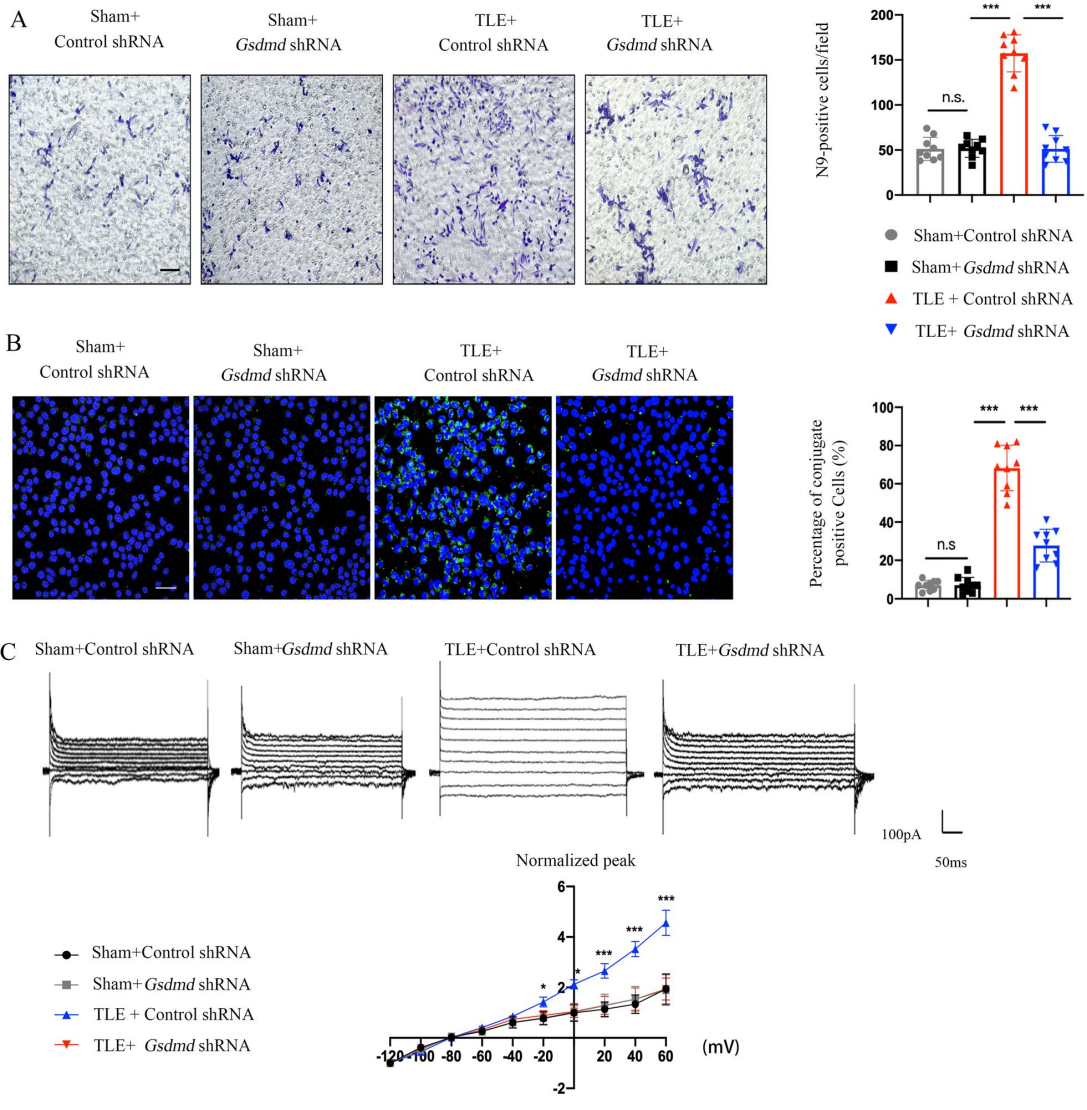
GSDMD knockdown attenuates the migration and phagocytic activity of microglia. **A** Images of crystal violet-stained N9 cells that migrated through the Transwell membrane and quantification of migrated N9 cells after treatment with KA or vehicle and *Gsdmd* shRNA or control shRNA(*n* = 9 in each group). **B** The phagocytosis of fluorescently labeled pHrodo BioParticle Conjugates by N9 cells was measured following treatment with vehicle, KA + control shRNA, or KA + *Gsdmd* shRNA. The bar graphs show the percentage of conjugates localized in DAPI^+^ cells (*n* = 9 in each group). **C** Current/voltage relationship at − 120 mV as a function of holding potential (mV) in the presence of KA or vehicle and after treatment with *Gsdmd* shRNA or control shRNA showing excessive inward rectification at positive holding potentials (*n* = 10 cells per group). In the representative images and statistical graph, the Y-axis represents the magnitude of the KCa3.1 amplitude and the normalized ratio, respectively. All data are presented as the mean ± SD; *P < 0.05, **P < 0.01, and ***P < 0.001

Since the impairment of phagocytosis led to the accumulation of apoptotic cells and the build-up of a detrimental inflammatory reaction, we sought to determine whether GSDMD knockdown-related augmentation of neuronal loss in TLE mice requires microglial migration and phagocytic activity. For this purpose, Transwell cultivation was subsequently performed to evaluate the migration and phagocytic activity of microglia. When the hippocampal neurons treated with KA in the lower chamber were damaged, the microglia in the upper chamber migrated to the permanent support membrane insert in the lower chamber. Then, using crystal violet staining to evaluate the number of migrated microglia, we found that the number of migrated microglia significantly decreased after GSDMD knockdown (Fig. 4A).

Next, the efficiency of N9 cells phagocytosis was determined by pHrodo BioParticles Conjugates and quantified using immunofluorescence staining. We found that the number of pHrodo BioParticles Conjugate consumed by N9 cells decreased significantly in the *Gsdmd* shRNA group; thus, compared to the control shRNA group, the phagocytotic capacity was lower (Fig. 4B). In the CNS, calcium-dependent potassium outward currents (KCa3.1 channels) are expressed by microglial cells and regulate cell phagocytic activity [25, 26]. After KA treatment, GSDMD knockdown reduced the amplitude of KCa3.1 channels (Fig. 4C), indicating that GSDMD knockdown weakened the phagocytic activity of microglia.

### GSDMD knockdown-enhanced accumulation of apoptotic neurons in TLE depends on phagocytic activity of microglia

**Fig. 5:**
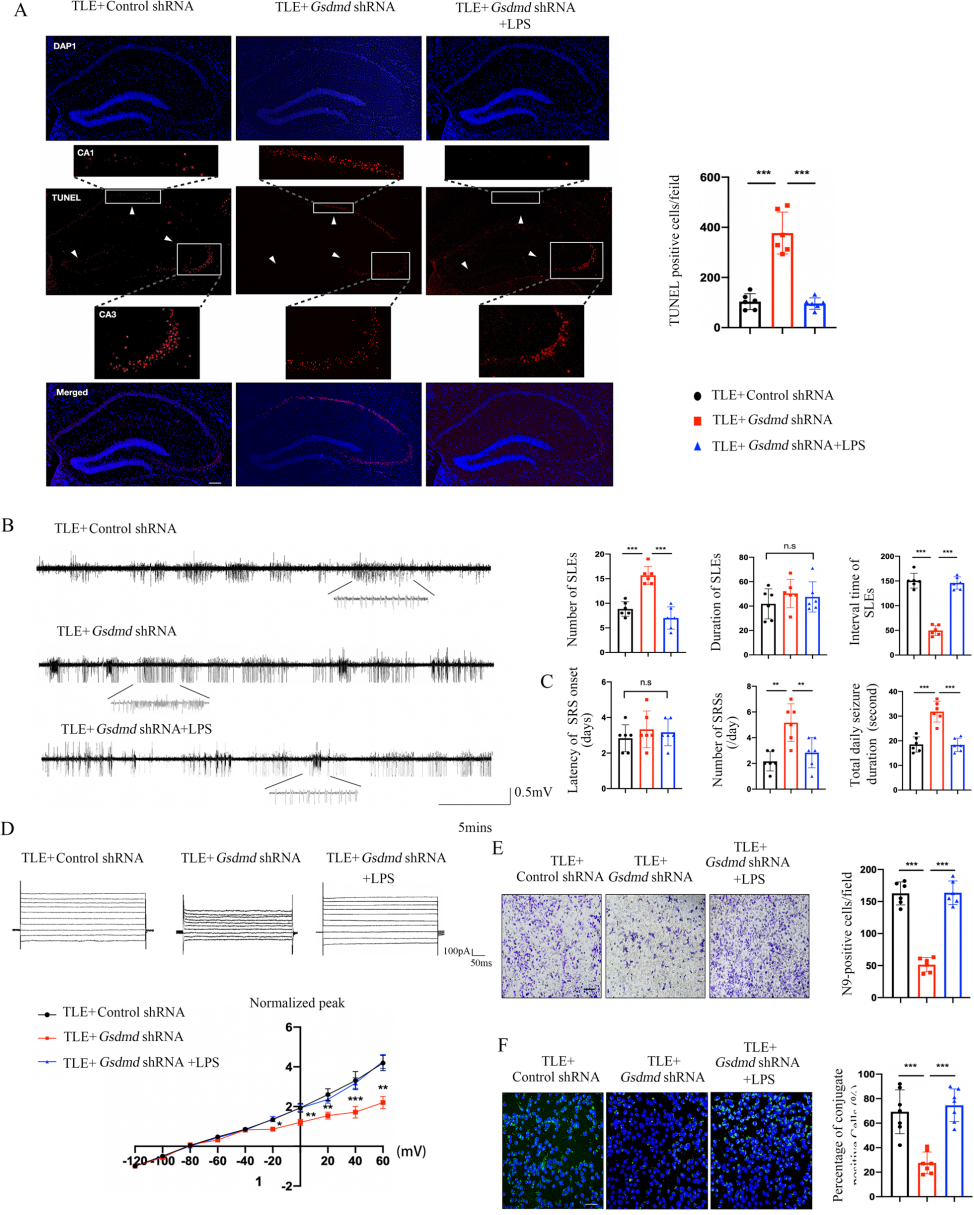
The GSDMD knockdown-mediated increase in apoptotic neuron accumulation in TLE depends on phagocytic activity of microglia. **A** Control shRNA or *Gsdmd* shRNA were injected into the hippocampus 3 weeks before KA administration and LPS treatment was given 24 h before KA administration. Scale bar = 100 µm. The bar graph shows quantitative analysis of the number of TUNEL^+^ cells in the hippocampus. (*n* = 6 mice per group). **B** Representative LFP signals in the TLE + control shRNA, TLE + *Gsdmd* shRNA and TLE + *Gsdmd* shRNA + LPS groups. The bar graphs show quantitative analysis of the number, duration, and interval of SLEs (*n* = 6 mice per group). **C** Quantitative analysis of the latency and number of SRSs and total daily seizure duration (*n* = 6 mice per group). **D** Current/voltage relationship at − 120 mV as a function of holding potential (mV) in N9 cells that received *Gsdmd* shRNA or control shRNA and were treated with LPS showing excessive inward rectification at positive holding potentials (*n* = 10 cells per group). **E** Images of crystal violet-stained LPS-activated N9 cells that migrated through the Transwell membrane in vitro and quantitative analysis of N9^+^ cells (*n* = 6). **F** Phagocytosis of fluorescently labeled pHrodo BioParticle Conjugates by N9 cells in the KA + control shRNA, KA + *Gsdmd* shRNA and KA + *Gsdmd* shRNA + LPS groups was measured. The bar graphs show the percentage of conjugates localized in DAPI^+^ cells (*n* = 7). All data are presented as the mean ± SD; *P < 0.05, **P < 0.01, and ***P < 0.001

To test the hypothesis that phagocytic activity of microglia is involved in GSDMD-mediated pyroptosis in TLE, microglia were activated with LPS. The transfection of primary hippocampal neurons was initially confirmed by detecting the expression of green fluorescent protein (GFP) encoded by an AAV vector using immunofluorescence staining (Additional file 1: Fig. S1B). The efficiency of GSDMD knockdown was confirmed by western blot at DIV7, and *Gsdmd* shRNA treated resulted in a significant reduction of GSDMD (Additional file 1: Fig. S1C). In vivo, LPS is injected into the bilateral hippocampus. We found that TUNEL-positive neurons increased after exposure to LPS-activated microglia in GSDMD knockdown mice, whereas the vehicle had no effect (Fig. 5A). In addition, LPS treatment alleviated seizure activity in GSDMD knockdown mice with TLE (Fig. 5B, C). Together, these results suggest that LPS-activated microglia diminish KA-induced seizure susceptibility and the accumulation of apoptotic neurons.

Next, we measured phagocytic activity of microglia after LPS treatment in recipients of *Gsdmd* shRNA or control shRNA. LPS treatment resulted in a significant increase in microglia that expressed KCa3.1 channels and P2Y_12_R in the control shRNA group but did not affect the *Gsdmd* shRNA group (Fig. 5C, D). In addition, we observed enhanced migration and phagocytic activity of microglia after LPS treatment by Transwell cultivation (Fig. 5E). Together, these results demonstrate that phagocytic activity of microglia significantly increased after GSDMD knockdown, suggesting that this enhanced the phagocytic activity of microglia may be involved in GSDMD-mediated pyroptosis in TLE.

### GSDMD knockdown attenuates phagocytic activity of microglia in a P2Y_12_R-dependent manner

**Fig. 6:**
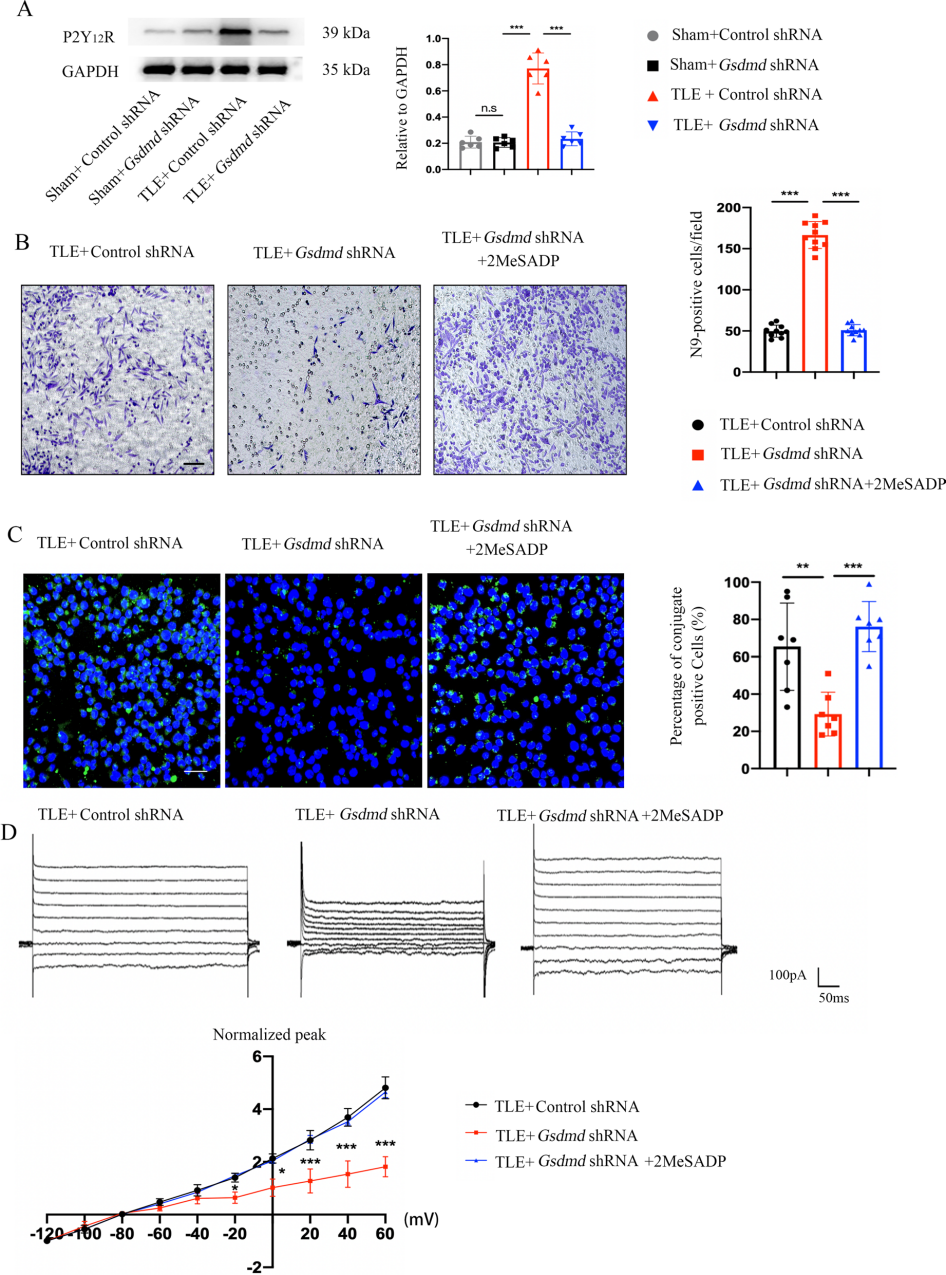
GSDMD knockdown attenuates phagocytic activity of microglia in a P2Y_12_R-dependent manner. **A **Western blot images and bar graphs showing the expression of P2Y_12_R in the hippocampus of mice with KA-induced TLE at 28 d after GSDMD knockdown (*n* = 6 mice per group). **B** The migration of N9 cells in the KA + control shRNA, KA + *Gsdmd* shRNA and KA + *Gsdmd* shRNA + 2MeSADP groups in vitro was measured by crystal violet staining (*n* = 10). **C **Images of fluorescently labeled pHrodo BioParticle Conjugates that were phagocytized by N9 cells. The bar graphs show the percentage of conjugates localized in DAPI^+^ cells (*n* = 7). **D** Current/voltage relationship in N9 cells that received *Gsdmd* shRNA or control shRNA and were treated with 2MeSADP showing excessive inward rectification at positive holding potentials (*n* = 10 cells per group). All data are presented as the mean ± SD; *P < 0.05, **P < 0.01, and ***P < 0.001

P2Y_12_R regulates microglial migration and phagocytic activity under physiological and pathological conditions [12]. We found that P2Y_12_R expression levels were decreased by western blotting in TLE mice after GSDMD knockdown. To examine further whether the phagocytic activity of microglia depends on P2Y_12_R, we evaluated the impact of a selective P2Y_12_R agonist, 2MeSADP, on the efficacy of microglial phagocytic activity by Transwell cultivation [27]. Crystal violet and immunofluorescence staining indicated that migration and phagocytic activity of microglia was enhanced after 2MeSADP treatment (Fig. 6B, C). In addition, 2MeSADP treatment significantly increased the amplitude of KCa3.1 channels in TLE mice after *Gsdmd* shRNA injection (Fig. 6D). These data suggest that the P2Y_12_R is necessary for phagocytic activity of microglia to provide neuroprotective effects.

## Discussion

This study provides novel evidence that GSDMD-mediated pyroptosis protects against KA-induced hippocampal apoptotic loss and seizure susceptibility. As documented here, inhibition of pyroptosis by GSDMD knockdown exacerbates the accumulation of dead neurons in the hippocampus and seizure behavior after KA treatment. Notably, GSDMD knockdown significantly attenuated KA-induced phagocytic activity of microglia but not astrocyte. In addition, LPS-activated microglia enhanced the neuroprotective effects of GSDMD-mediated pyroptosis, suggesting that microglia are needed to mediate the beneficial effects of GSDMD-mediated pyroptosis. Finally, the P2Y_12_R agonist 2MeSADP enhanced the migration and phagocytic activity of microglia, indicating that GSDMD knockdown weakened the phagocytic activity of microglia by P2Y_12_R. Our results indicate that P2Y_12_R-mediated phagocytic activity of microglia may be involved in the neuroprotective effects of GSDMD-mediated pyroptosis in KA-induced TLE mice.

Considerable evidence points to GSDMD-mediated pyroptosis as an important role of TLE [4]. Reportedly, elevated levels of the NOD-like receptor pyrin domain-containing protein 1 (NLRP1) inflammasome and caspase-1 were observed in the hippocampal neurons of TLE patients, and inhibition of NLRP1 or caspase-1 alleviated pyroptosis-related neuronal loss and the severity of seizures in an animal model of TLE [4]. Notably, inhibition of GSDMD-mediated pyroptosis by dimethyl fumarate attenuated the severity of seizures and astroglial damage in KA-induced epileptic mice. Overall, the conclusion about the relationship between pyroptosis and TLE is not consistent, which indicates the heterogeneity of TLE and the complexity of the immune microenvironment [28]. In fact, accumulated evidences showed that the effect of GSDMD-mediated pyroptosis in the CNS is complicated. Inhibiting GSDMD has exhibited promising neuroprotective effects in some neurological disorders. McKenzie et al. found a significantly increased GSDMD level in the microglia in the multiple sclerosis humans and animals, while inhibition of GSDMD could dramatically decrease inflammatory factors release, reduce axonal damage and behavioral impairment [7]. Similar findings have been reported in Parkinson’s disease (PD) [29]. In contrast, part of studies showed the protective role of GSDMD-mediated pyroptosis in neurons, while inhibiting GSDMD can lead to neurological abnormalities [30]. The complex role of GSDMD-mediated pyroptosis in the CNS may be related to the different cell types that highly express GSDMD in different pathological disorders or physiological conditions [31]. However, no direct evidence has confirmed whether GSDMD-mediated pyroptosis is the cause or consequence of hippocampal neuronal loss, which would explain how GSDMD-mediated pyroptosis causes detrimental or beneficial effects in TLE. Nevertheless, in line with our previous findings, we show here that GSDMD knockdown exacerbates the hippocampal neuronal loss induced by KA. These results, together with the data showing that dead hippocampal neurons are augmented after GSDMD knockdown in KA-treated mice, demonstrate the protective role for GSDMD in TLE.

Our previous data demonstrate that GSDMD inhibition leads to a transition from pyroptosis to apoptosis. Similarly, we found that GSDMD knockdown aggravated KA-induced hippocampal neuronal apoptosis by TUNEL staining. To determine the underlying mechanisms by which GSDMD knockdown triggers hippocampal neuronal apoptosis in response to KA, we examined phagocytic activity of microglia in the hippocampus. Microglial phagocytic impairment leads to the accumulation of apoptotic cells and contributes to the development of an inflammatory response, which can worsen KA-induced hippocampal neuronal loss after GSDMD knockdown [3]. In support of this view, we found that knockdown of GSDMD led to reduced efficiency of phagocytic activity of microglia and promoted microglial M1 polarization and local inflammation in the hippocampus. Similarly, the protective role of GSDMD against KA was observed in LPS-treated mice. In addition, our results showed that the number of astrocytes was not significantly affected in KA-treated mice after GSDMD knockdown compared with control shRNA-treated mice. Reportedly, while nonprofessional phagocytes (astrocytes or neuroblasts) become engaged in phagocytosis in epilepsy, they engulf only a small proportion of apoptotic cells compared to microglia [3]. Although nonprofessional phagocytes are recruited in an attempt to compensate for phagocytic activity of microglia, the cell type that contributes the most to phagocytic activity in the hippocampus remains the impaired microglia. Therefore, we hypothesize that GSDMD can provide neuroprotective effects by promoting phagocytic activity of microglia.

P2Y_12_R, a microglial purinoceptor, is essential for microglia–neuron interactions, senses the extracellular ADP released from damaged neurons and mediates microglia-directed migration and phagocytic activity [32]. This result suggests that the activation of P2Y_12_R may promote phagocytic activity of microglia in TLE. Accordingly, we found that 2MeSADP treatment significantly enhanced the migration and phagocytic activity of microglia after GSDMD knockdown. Reportedly, P2Y_12_ was not expressed in other glial cells, such as astrocytes [33], which also explains why no change in the number of astrocytes was observed after GSDMD knockdown. Therefore, we hypothesize that GSDMD can provide neuroprotective effects by enhancing P2Y_12_R-mediated phagocytic activity of microglia in TLE. This possibility should be further investigated, and the possible multifaceted functions of microglia cannot be excluded.

In summary, our data suggest that GSDMD-mediated pyroptosis orchestrates phagocytic activity of microglia and protects against the neuronal loss induced by KA. Therefore, we propose that the modulation of phagocytic activity of microglia is a novel and yet unexplored therapy to accelerate functional brain recovery in TLE.

### Supplementary Information

**Additional file 1: Figure S1.** Verification of the knockdown effect of *Gsdmd* shRNA. A. Representative immunoblots and quantitative analysis of full-length GSDMD in the hippocampus on day 28 after KA injection (*n* = 5 per group). B. Expression of GFP encoded by AAV carrying *Gsdmd*-shRNA-GFP in primary hippocampal neuronal. C. Representative immunoblots and quantitative analysis of full-length GSDMD from neuronal lysates (*n* = 6 per group). All data are presented as the mean ± SD; ***P < 0.001.

## Data Availability

The datasets used and/or analyzed are available from the corresponding author on reasonable request.
