# Supplementary figures and images for: GSDMD knockdown attenuates phagocytic activity of microglia and exacerbates seizure susceptibility in TLE mice
Source: J Neuroinflammation. 2023 Aug 23;20:193. doi: 10.1186/s12974-023-02876-w (PMC10464294; doi:10.1186/s12974-023-02876-w)

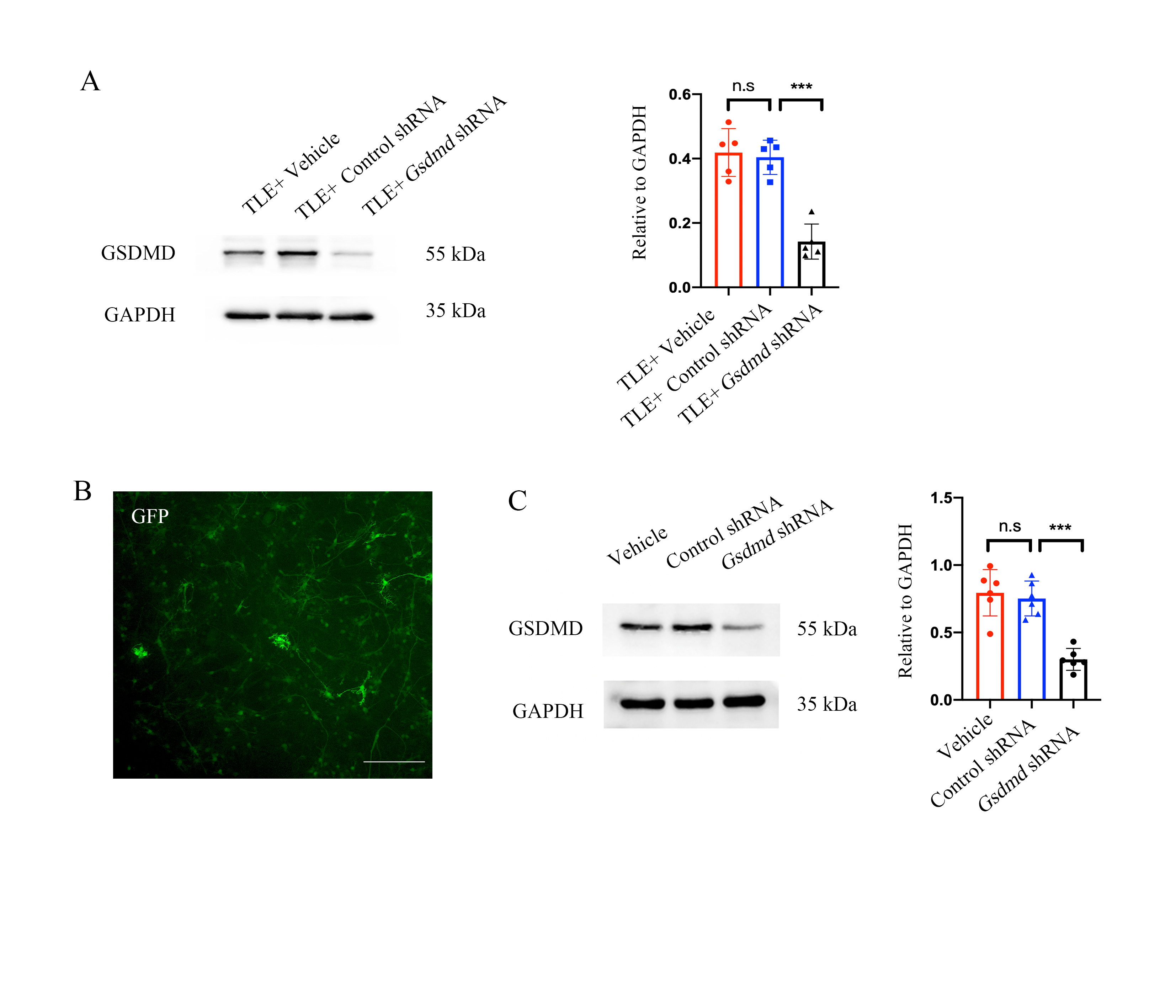

Supplement: Supplementary file 1 — Additional file 1: Figure S1. Verification of the knockdown effect of Gsdmd shRNA. A. Representative immunoblots and quantitative analysis of full-length GSDMD in the hippocampus on day 28 after KA injection (n = 5 per group). B. Expression of GFP encoded by AAV carrying Gsdmd-shRNA-GFP in primary hippocampal neuronal. C. Representative immunoblots and quantitative analysis of full-length GSDMD from neuronal lysates (n = 6 per group). All data are presented as the mean ± SD; ***P < 0.001. [file 12974_2023_2876_MOESM1_ESM.tif]
